# A pilot randomized controlled trial of the ABCs of SLEEPING mHealth intervention for parents of school-aged children with insomnia symptoms

**DOI:** 10.3389/frsle.2025.1665671

**Published:** 2026-01-29

**Authors:** Anastasija Jemcov, Penny Violet Corkum, Isabel M. Smith, Sean P. Mackinnon

**Affiliations:** 1 Department of Psychology & Neuroscience, Dalhousie University, Halifax, NS, Canada; 2 Dalhousie University, IWK Children's Hospital, Halifax, NS, Canada

**Keywords:** child, healthy sleep practices, insomnia symptoms, mHealth, mobile applications, pilot RCT, school-aged children, sleep intervention

## Abstract

**Introduction:**

Sleep is important for overall functioning; thus, parents should have access to effective sleep intervention for their children's insomnia. Mobile health interventions (mHealth) are increasingly popular partly due to their accessibility. Currently, no evidence-based sleep intervention apps are available for parents and their school-age children. Our research team developed the ABCs of SLEEPING intervention to address this gap.

**Method:**

The current study used a modified version based on feedback from a feasibility study which found reasonable acceptability and promising preliminary effectiveness but lower fidelity than expected (i.e., not daily use). The current study examined preliminary effectiveness using randomized controlled trial (RCT) methodology for subjective (sleep habits, insomnia severity, behavioral functioning) and objective sleep variables, and examined recruitment data to inform practices for a future RCT. Participants were 28 parents of typically developing children with parent-reported sleep problems, randomized to a treatment or control group. Data were analyzed using descriptive statistics and analysis of covariance (ANCOVA).

**Results:**

Recruitment rate was 70%, dropout rate was 30%, and estimated sample size for an RCT was 118. A small effect of the intervention improving sleep habits, daytime functioning, and insomnia severity, and no statistically significant effect for objectively measured sleep were demonstrated.

**Discussion:**

These results can be used to modify the intervention and to prepare for a large-scale effectiveness study. As an accessible mHealth intervention for parents of school-aged children with insomnia, the ABCs of SLEEPING app has the potential to address an existing treatment gap.

## Introduction

Sleep is essential for children's growth and development. Unfortunately, sleep problems are widespread among school-age children in Canada, with one out of five children in the general population experiencing a significant sleep problem (Calhoun et al., 2014). The most common sleep problems experienced by school-age children are difficulties falling/staying asleep and early morning awakenings. When these symptoms are chronic and cause clinically significant distress, they are grouped as insomnia. Insomnia symptoms are associated with increased behavioral difficulties, emotional dysregulation, and poorer academic performance (Chaput et al., 2016; Calhoun et al., 2017). Given the negative consequences of insomnia symptoms, it is important that children experiencing them receive timely and effective sleep interventions.

A range of sleep interventions exist, including sleep psychoeducation (i.e., teaching sleep knowledge to children and their parents/caregivers and addressing misinformation), making changes to sleep habits (e.g., removing electronics from bedroom, adjusting timing of sleep/wake times, changing location of sleep), using specific behavioral interventions (e.g., bedtime fading), and sleep medication (e.g., non-prescription like antihistamines and melatonin and prescription medications such as alpha-receptor agonists; Thomas et al., 2014). All of these interventions have their place within a stepped approach to sleep treatment (Rigney et al., 2021). Specifically, less intensive and more economical approaches (sleep education, changing sleep habits) should be tried first, with those that are more intensive and costly (e.g., sleep programs with specific behavioral interventions like bedtime fading) implemented if needed. Only if the sleep education and behavioral interventions are tried without adequate improvements to sleep problems should medication be trialed in combination with behavioral strategies. This is because medication alone does not improve sleep problems over the long term, as its impacts tend to be short lived (Mack and Rybarczyk, 2011; Riemann and Perlis, 2009). Moreover, evidence for the safety and tolerability of medications in children is limited, and their use in the treatment of pediatric sleep problems is not approved by the American Food and Drug Administration (FDA) (Mindell et al., 2006; Pelayo and Dubik, 2008; Lunsford-Avery et al., 2020; Bock et al., 2016). These approaches to sleep treatment are organized in a stepped care model. Specifically, step 1 would be teaching sleep information, addressing inaccurate information and parental beliefs about sleep. Step 2 would be making changes to sleep habits (previously referred to as sleep hygiene). Behavioral interventions (e.g., cognitive behavioral therapy focused on insomnia) would be step 3. Finally, step 4 would include non-prescription medications such as antihistamines and prescription medications such as trazodone.

A prominent issue in delivering sleep treatment has been accessibility barriers. A myriad of studies demonstrate that costs (time and financial), transportation, geographical location, schedule/time demands, psychosocial factors (e.g., parental stress), lack of available programs and interventions or awareness of them, and healthcare providers (HCPs) untrained in treating insomnia are all significant barriers to receiving and engaging with sleep treatment (Whittall et al., 2021; Tan-MacNeill et al., 2020; Billings et al., 2021; Paterson et al., 2019; Roberts and Ulmer, 2024; Stinson et al., 2006; Boerner et al., 2015). Historically, efforts have been made to make interventions more accessible via alternative electronic forms of delivering care (e.g., videoconferencing, internet, and website interventions; Corkum et al., 2018; Tan-MacNeill et al., 2020; Wosik et al., 2020; Santarossa et al., 2018; Buckman et al., 2021; Kreps and Neuhauser, 2010).

Smartphone applications (“apps”) are a newer form of intervention delivery, often referred to as mobile health (mHealth; Marcolino et al., 2018). Although currently no empirically supported smartphone apps address insomnia for school-age children, studies have demonstrated the acceptability and effectiveness of smartphone apps changing sleep outcomes in infants, adolescents, and adults (Yoshizaki et al., 2020, 2023; Quante et al., 2019; Rayward et al., 2020). Apps can provide a convenient and flexible way for people to access mental health resources and support. This is particularly important for individuals who may have limited access to traditional face-to-face treatment due to geographical and financial barriers. mHealth interventions have the potential to improve treatment access for Canadians who have a smartphone (i.e., approximately 85% have access for personal use; Statistics Canada, 2021).

To address this resource gap, our research team has developed a smartphone app called the ABCs of SLEEPING. This intervention was developed to provide parents and their school-age children (6 to 12 years old) with evidence-based knowledge and recommendations to improve their children's sleep habits and insomnia symptoms. The ABCs of SLEEPING is a mnemonic that draws attention to the important factors that must be considered for healthy sleep practices for children: *A*ge-appropriate Bedtimes and wakeup times with Consistency, Schedules and routines, Location, Exercise and diet, no Electronics in the bedroom or before bed, Positivity, Independence, and Great sleep (Bessey et al., 2013). To validate the use of this mnemonic, Allen et al. (2016) conducted a review of the evidence evaluating the empirical support for each of the ABCs of SLEEPING recommendations.

The development of the ABCs of SLEEPING app has been guided by a user-centered approach, as outlined by Lyon and Koerner (2016). This approach involves continuously engaging end users such as parents and HCPs throughout the development and design phases to ensure that the content and recommendations address end users' needs and desires. Many steps were taken to ensure that the ABCs of SLEEPING intervention was liked by parents and implemented as intended, in addition to demonstrating evidence of intended effect (i.e., improving sleep habits and insomnia symptoms). The first was a usability study that used Morville's User Experience Honeycomb model (Morville and Sullenger, 2010) to examine whether parents and HCPs viewed the intervention as useful, usable, desirable, valuable, accessible, credible, and findable (Howlett et al., 2020). Parents considered the intervention to be highly usable, desirable, accessible, and credible. Parent and HCP participants gave feedback regarding how the intervention could be modified to meet their needs. Feedback from HCPs regarded adding a feature to share/receive data and the idea that some families might require more support than the ABCs of SLEEPING intervention could offer. Parent feedback included the recommendation to centralize all components of the intervention in one package—in the initial version components were provided as an online survey (check-in) and portable document format files (PDFs; sleep reports, sleep tips). Additional feedback from parents was to provide a method of prioritizing healthy sleep practice areas to focus on, adding features and making aesthetic changes (e.g., including frequently asked questions, open text fields at check-in, adding more color), and modifying the sleep tips to be less overwhelming, age-specific, and personalized. Modifications were made to address their feedback (i.e., centralization of all components in a smartphone app, color-coding and star system for prioritization, addition of color, adjustment of sleep tips based on feedback, for example).

Following this, we used Bowen et al. (2009) feasibility framework to understand important aspects of feasibility (Jemcov et al., 2021). This framework provides a guideline with approaches such as “can it work” (e.g., is there evidence that the intervention might work with the intended population?), “does it work” (e.g., is there evidence that the intervention may be efficacious or effective under ideal or actual circumstances?) and “will it work?” (e.g., will the intervention work in a real-life context, such as with populations that might adopt it in practice?) Additionally, this framework identifies important areas of feasibility that may be explored. Using the “can it work?” approach, we examined acceptability (i.e., satisfaction and suitability ratings of the intervention), fidelity (i.e., did parents use the intervention as intended by the researchers, which was daily use of the sleep tips), and whether there was evidence of preliminary effectiveness (i.e., testing before/after changes in the intended outcomes of the intervention in a limited way). Examining preliminary effectiveness is not equivalent to well-powered hypothesis testing; rather, our goal was to look for evidence of the intended effects of the intervention (Bowen et al., 2009).

The feasibility study revealed that the ABCs of SLEEPING intervention suggested preliminary effectiveness with a medium effect size based on subjective measures of sleep outcomes (i.e., sleep habits and insomnia severity improved following intervention use) and reasonable acceptability, but fidelity to the intervention was not as expected in that parents did not use the sleep tips daily (Jemcov et al., 2021). Parent feedback indicated that sleep tips required further refinement and more guidance regarding how to prioritize the sleep tips. Additionally, parents reported not remembering to use the sleep tips daily and noted that a reminder feature would have been helpful. Two features were added to the ABCs of SLEEPING intervention to address this feedback; sleep tips were refined (e.g., resources added) and a to-do list (i.e., a feature that enables parents to prioritize which sleep tips to start with) and a reminder feature (i.e., push notification) were added.

Conducting a pilot RCT was deemed to be the next crucial step ahead of a full-scale RCT in the development and evaluation phase of this app. A pilot RCT allows for collecting feasibility data to inform a full-scale RCT. Specifically, the recruitment potential, participant retention, and dropout rate can be examined while utilizing the RCT methodology (Bond et al., 2023; Leon et al., 2011). Additionally, a priori calculations can be made with these data to estimate sample size for the full-scale RCT. Finally, preliminary effectiveness can be examined with increased experimental control through randomization, and the use of an objective sleep measure (actigraphy) increases rigor. As such, this study's first aim was to provide estimates for a RCT, specifically, recruitment potential, participant retention, dropout rate, and sample size. The second aim was to examine, using the “does it work?” approach, preliminary effectiveness by evaluating, for each group, differential change from before intervention to after intervention for subjective measures of sleep habits, insomnia severity, and daytime functioning and for an objective measure of sleep efficiency (SEF) and sleep onset latency (SOL). Given these aims the research questions were as follows:

(1) What are the recruitment potential, retention, dropout rate, and sample size estimates for a future full-scale RCT?
(2) Do parents in the treatment group report improvements based on subjective measures of their children's sleep habits, insomnia symptom severity, and behavioral functioning compared to a waitlist control group?
(3) Do the children of the parents in the treatment group show improvements on objective actigraphy sleep measures (i.e., SOL and SEF), compared to a waitlist control group?

## Method

### Participants

Twenty-eight parents of typically developing English-speaking children were recruited using social media advertisements on Instagram and Facebook. The children of the participating parents were required to have at least one of three insomnia symptoms (i.e., difficulty falling asleep, staying asleep, and/or early morning awakenings) and did not require a diagnosis of insomnia. Insomnia symptoms were measured using the Pediatric Insomnia Severity Index (PISI), which is used to describe insomnia severity in our sample but not as an eligibility criterion. Inclusion criteria were that parents (a) resided in Canada, (b) had internet/computer access and an email to complete online questionnaires, and (c) were comfortable reading and writing in English. Their children needed to (a) be between the ages of 6 and 12 years, (b) not have a diagnosis of a mental or physical health disorder that would significantly impact daily functioning, and (c) have one or more insomnia symptoms with no sleep apnea: difficulties falling asleep, staying asleep, and/or early morning awakenings with the inability to fall back asleep. Participants in middle childhood (ages 6–12) were studied because this represents a period when sleep problems are both common and responsive to behavioral interventions, as well as a time when parents remain closely involved in bedtime routines. Ethical approval for this study was obtained through the IWK Health Centre Research Ethics Board (REB; 1027369).

### Intervention

Participants randomized to the treatment group received 1-month access to the ABCs of SLEEPING intervention that consisted of three components. The first component is the sleep check-in, which is a sleep assessment that parents complete when opening the app. Parents answer questions about their children's sleep habits, and their answers algorithmically produce the second and third components, the sleep report card and sleep tips. Specifically, an algorithm automatically produces a sleep report that provides prioritized sleep tips using a star- and color-coded system. The sleep report card provides an overview of the child's sleep habits organized by the ABCs of SLEEPING mnemonic (see introduction). Parents are given ratings using a three-star system to identify healthy sleep practices they are performing well (three stars), those in which they need some work (two stars), and those requiring more work (one star). The sleep tips are also organized by the mnemonic, providing parents with sleep recommendations to make changes to areas identified as needing work. The areas needing no work are coded as green, those needing some work are yellow, and those needing the most work are red.

### Measures

#### Eligibility questionnaire (EQ)

We designed the 14-item EQ to assess the study's inclusion and exclusion criteria. Potential participants provided “yes” or “no” answers to each item. This questionnaire was fully automated with two outcomes: pass or fail eligibility screening.

#### Demographic questionnaire (DQ)

Participants completed the DQ during the pre-intervention period to describe the study sample. The DQ includes 24 or 29 items, depending on whether there is a partner/spouse. Demographic information (e.g., age, sex) was collected about participating parents, partners/spouses (if applicable), and their children. Items were compiled from the Statistics Canada (2011) as well as the Canadian Census.

#### Children's sleep habits questionnaire (CSHQ)

Participants completed the 52-item CSHQ at the pre-intervention and post-intervention periods. This questionnaire collects information regarding the frequency with which children engaged in certain sleep-related behaviors over the last typical week (e.g., child goes to bed at the same time each night) on a scale of 3 (“usually”) (five to seven times) to 1 (“rarely”) (0–1 time). This questionnaire uses select items to derive the total sleep disturbance score (which was used in this study). The total sleep disturbance score ranges from 33 to 99, and a score of 41 or higher indicates clinically significant sleep problems (Owens et al., 2000a,b). The questionnaire was designed for school-age children and yields scores across eight subscales: bedtime resistance, sleep resistance, parasomnia, sleep disordered breathing, night wakings, daytime sleepiness, sleep anxiety, and sleep onset delay, as well as a summative total sleep disturbance score. The CSHQ demonstrates good sensitivity (0.80) and specificity in prior research (0.72) (Owens et al., 2000a,b).

#### Bedtime routines questionnaire (BRQ)

Before and after intervention, participants completed the 31-item BRQ, which measures children's bedtime routines (e.g., read/listen to a story, watch TV, brush teeth) on a scale ranging from 1 (“almost never”) to 5 (“nearly always”). The BRQ yields a summative score for the consistency of a child's weekday and weekend bedtime routines, with higher scores indicating greater consistency; scores can range from 31 to 155. We utilized the total summative score for both weekday and bedtime routines. Overall, the BRQ demonstrates internal consistency in past research (0.69–0.90) (Henderson and Jordan, 2010).

#### Strengths and difficulties questionnaire (SDQ)

Parents completed this 25-item measure during the pre- and post-intervention periods. The SDQ assesses prosocial (e.g., considerate of other's feelings) and challenging behaviors (e.g., often loses temper) of children as reported by their parents. A summative total difficulties score, which was used in this study, includes emotional symptoms, behavioral problems, hyperactivity/inattention, and peer relationship problems, but the prosocial behavior scale is not included. Each item is measured on a scale ranging from 0 (“not true”) to 2 (“certainly true”), with possible total difficulties scores ranging from 0 to 40 as 20 items are scored (Goodman, 1997). Reliability scores confirmed strong support for internal consistency in a representative Canadian sample of school-age children and adolescents (Hoffman et al., 2020).

#### Pediatric insomnia severity index (PISI)

Participants completed the seven-item PISI measure during the pre- and post-intervention periods (Byars and Simon, 2014). The PISI assesses insomnia symptoms using a parental report of sleep maintenance problems, daytime sleepiness, and nocturnal sleep duration with children ages 4–10 years old. Given that the sample did not include any children over the age of 10, the adolescent version (i.e., PISI—Adolescent; PISI-A), which is validated for age 11 and above, was not required or administered. Items (e.g., “my child takes longer than 30 min to fall asleep after going to bed”) are rated on a 6-point scale ranging from 0 (“never”) to 6 (“always”), and a total summative score can be calculated with a minimum of 0 and a maximum of 30 using the first five items (Angelhoff et al., 2020). The total summative score was used to describe the severity of insomnia in this sample, as well as to assess changes in insomnia severity. The PISI has high internal consistency between items measuring sleep onset problems and sleep maintenance problems in past research (Byars et al., 2017).

#### Actigraphy

An actigraph is a watch-like device, typically worn on the non-dominant wrist, that collects movement information using an accelerometer. Previous studies showed that the software algorithms used to analyze actigraphy data provide valid and reliable estimates of when participants are asleep and awake, as well as variables such as sleep latency and duration (Acebo et al., 1999; Tryon, 2004). We used the Philips Actiwatch 2, and the variables of interest were SOL (how quickly a child falls asleep once in bed and ready to sleep) and SEF (the percentage of time scored as asleep during the period from the time the child is in bed and ready to fall asleep to the time the child is out of bed for the day). Actigraph data were collected during the pre- and post-intervention periods for a minimum of five corresponding nights of sleep diary and actigraphy, considered to provide a valid estimate of typical sleep (Sadeh, 2008). In our sample, participants wore the actigraph approximately 6 days out of 7 (*M* = 6.07, SD = 0.87).

#### Sleep diary (SLD)

Sleep diary data were collected to provide contextual markers necessary for valid actigraphy scoring. The SLD was completed online by parents who reported on their children's sleep for 1 week during each of the pre- and post-intervention periods. Information such as what time their child was “down for the night,” “up for the day,” and anything unusual that happened during the day that could impact sleep was collected. This is information necessary to score the actigraphy data. The minimum requirement was 5 nights of corresponding SLD and actigraphy data. The diary took approximately 5 min to complete daily. Parents were provided with a paper template in their study package if they preferred to write down their entries and later document their sleep diaries online.

### Procedure

Potential participants expressed interest in the study by either emailing the research coordinator or following a link in the study's social media advertisements that led parents to the online EQ form to assess eligibility. Upon completing the EQ, parents were automatically informed if they were eligible or not. If eligible, they were directed to the consent form, which they completed online. Next, participants were emailed to arrange for a telephone call that was subsequently completed to review the consent form before proceeding. Parents were then sent an email with a REDCap link containing the pre-intervention measures (i.e., DQ, CSHQ, BRQ, PISI, and SDQ). At the same time, participants were mailed a study package containing an actigraph and instructions for using it and a paper copy of the SLD (the SLD paper copy was intended for parents wanting to take notes on it to later input the information into REDCap). Parents were instructed to have their child wear the actigraph for 1 week continuously and to complete a SLD each night that the actigraph was worn. After this week, the actigraph was sent back. Once the actigraph was returned and all the pre-intervention measures were confirmed completed, this concluded the pre-intervention period. Parents were then randomized to either the treatment or waitlist control group. Block randomization (defined as having a preset value of participants to be assigned to each group to ensure balance; Kang et al., 2008) was used with blocks of four. Those randomized to the treatment group were given access to the intervention and instructed to use the sleep tips daily for 1 month. Following this month, participants were sent an actigraph again, along with a link to the SLD for the post-intervention period. The same procedures were implemented for the post-intervention period as the pre-intervention period, except no DQ was administered. After the post-intervention period, participants were thanked and given a $20 Amazon.ca gift card. Upon completing the post-intervention measures, waitlist control group participants were given access to the intervention for 1 month, but no additional data were collected (as noted previously).

### Data analytic approach

**Figure 1:**
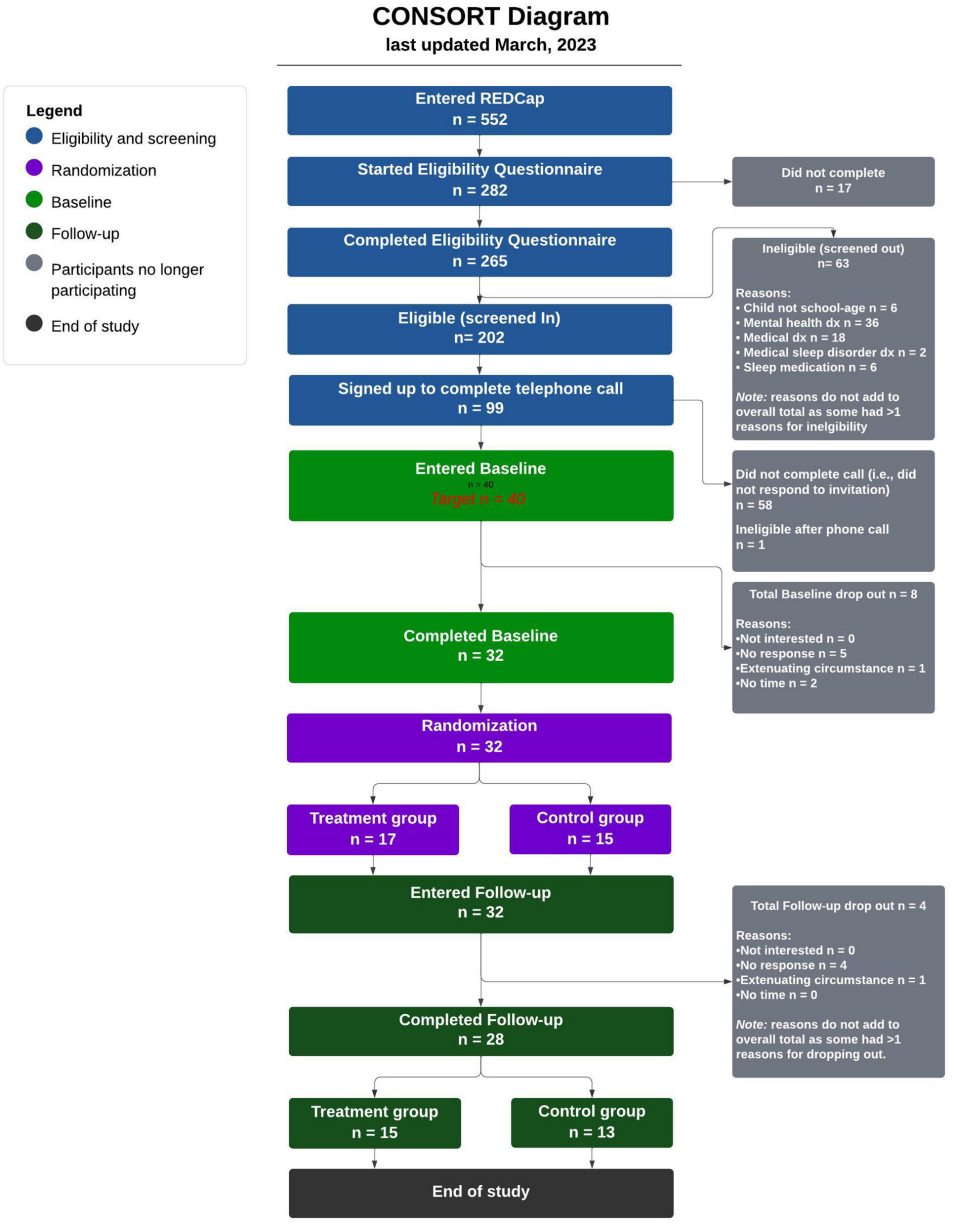
The ABCs of SLEEPING pilot RCT CONSORT diagram.

Participant flow through the study was tracked and compiled in a CONSORT diagram (Figure 1), which describes participant flow through the study. These descriptive statistics were used to estimate parameters needed for a full-scale RCT. To examine preliminary effectiveness, one analysis of covariance (ANCOVA) was conducted for each of the study's subjective and objective outcome measures (six ANCOVAs total), with pre-intervention scores used as the covariate. Prior to conducting the analyses, the data were screened to examine whether ANCOVA assumptions were met. Normality of residuals was assessed by reviewing Q–Q plots, and multivariate outliers were assessed with Cook's distance (with values >0.50 inspected for high influence). Additionally, assumption of equal variance was assessed using Levene's test. Those variables violating the equal variance or normality assumption were assessed with sensitivity checks by reanalyzing the data with robust ANCOVAs to examine whether the original ANCOVA results held. Data from the non-robust ANCOVAs were reported if no differences were observed after completing the robust ANCOVA. Robust ANCOVA results are reported in the notes of the respective tables. Given the small sample size for both subjective and objective data, we examined ω^2^ effect sizes due to their decreased bias with smaller sample sizes.

Given that participants were randomly assigned to the treatment and waitlist control groups, independence of treatment and covariate were confirmed by design; thus, there was no need to compare treatment vs. control groups on pre-intervention measures (Senn, 1994). A sensitivity power analysis was conducted in G^*^Power for ANCOVA with the following parameters: power = 0.80, α = 0.05, d*f* = 1, groups = 2, covariate = 1. The sensitivity power analysis revealed that with a sample size of 28, the largest effect size that could be detected would be *f* = 0.55 (i.e., $\eta_{p}^{2}$ = 0.23).

## Results

### Participants and recruitment potential

The flow of participants through the study can be seen in the CONSORT diagram (Figure 1). In total, 552 participants entered the study by clicking a REDCap link provided through study advertisements (e.g., social media posts). Of these 552 parents, the EQ was completed by 265, of which 202 were eligible to enroll in the study. The most common reason for ineligibility noted was a diagnosis of a significant disorder related to either mental or physical health (all reasons for ineligibility are provided in Figure 1). Of those eligible, 99 were responsive to an email requesting they complete a telephone call, but only 41 completed the phone call with the study's researcher (AJ). One participant was ineligible after this phone call as a result of disclosing that their child was taking sleep medication. Thus, 40 participants entered the study's pre-intervention period, and 32 completed this phase and were randomized to either the treatment group (*n* = 17; 10 females and 7 males) or the waitlist control group (*n* = 15; 11 females and 4 males). Two from each group (four in total) dropped out as a result of being nonresponsive to emails or study procedures. As such, 28 participants completed the post-intervention period, which concluded their participation. Our final sample consisted of 28 parents of typically developing English-speaking children, 15 of whom were in the treatment group (8 females and 7 males), and 13 were in the waitlist control group (9 females and 4 males). Insomnia severity at pre-intervention for the whole child sample (*n* = 37) was *M* = 15.30, SD = 4.16, out of a maximum of 30. The insomnia severity at pre-intervention for the final sample (*n* = 28) was *M* = 15.70, SD = 4.43. (Please see Tables 1 and 2 for a detailed breakdown of participant characteristics at baseline and post-test, respectively.) Our final sample of participating parents' children were mostly White (71%) and female (64%), and parents were predominantly highly educated (85% had a bachelor's degree or higher) biological mothers (82%).

**Table 1:** Baseline demographic and descriptive information for all participating parents and children.

**Parent demographics (Tx Group; *N =* 17)**		**Parent demographics (Ctrl Group; *N =* 15)**	
**Relationship to child**	***N*** **(%)**	**Relationship to child**	***N*** **(%)**
Father	3 (18.0)	Father	2 (13.0)
Mother	14 (82.0)	Mother	13 (87.0)
**Current employment status**		**Current employment status**	
Full Time	13 (76.0)	Full Time	12 (80.0)
Part Time	4 (24.0)	Part Time	0 (0.0)
Homemaker	0 (0.0)	Homemaker	3 (20.0)
**Estimated household income**		**Estimated household income**	
$29,999 and under	0 (0.0)	$29,999 and under	0 (0.0)
$59,999–$30,000	0 (0.0)	$59,999–$30,000	0 (0.0)
$79,999–$60,000	4 (24.0)	$79,999–$60,000	2 (13.0)
$80,000–$124,999	6 (35.0)	$80,000–$124,999	6 (40.0)
$125,000 and over	7 (41.0)	$125,000 and over	7 (47.0)
**Type of community of residence**		**Type of community of residence**	
City	12 (71.0)	City	9 (60.0)
Town	4 (24.0)	Town	3 (20.0)
Rural	1 (5.0)	Rural	3 (20.0)
**Highest level of education**		**Highest level of education**	
Community college/high school	2 (12.0)	Community college/high school	2 (13.3)
Bachelor's degree/undergraduate	8 (47.0)	Bachelor's degree/undergraduate	6 (40.0)
Master's degree	2 (12.0)	Master's degree	6 (40.0)
Degree in medicine	5 (29.0)	Degree in medicine	1 (6.7)
**Child demographics (Tx group;** ***N** =* **17)**		**Child demographics (Ctrl group;** ***N** =* **15)**	
**Child sex**	***N*** **(%)**	**Child sex**	***N*** **(%)**
Male	7 (41.2)	Male	4 (27.0)
Female	10 (58.8)	Female	11 (73.0)
Child Mean Age in Years (*SD*)	8.53 (1.84)	Child Mean Age in Years (*SD*)	8.47 (1.46)
**Child's ethnic or cultural heritage**		**Child's ethnic or cultural heritage**	
White	13 (76.4)	White	11 (73.4)
Indigenous	1 (6.0)	Indigenous	2 (13.3)
Other	3 (17.6)	Other	2 (13.3)

Tx, Treatment; Ctrl, Control.

**Table 2:** Post-test demographic and descriptive information for all participating parents and children.

**Parent Demographics (Tx Group; *N =* 15)**		**Parent Demographics (Ctrl Group *N =* 13)**	
**Relationship to child**	***N*** **(%)**	**Relationship to child**	***N*** **(%)**
Father	3 (20.0)	Father	1 (7.7)
Mother	12 (80.0)	Mother	12 (92.3)
**Current employment status**		**Current employment status**	
Full Time	11 (73.3)	Full Time	10 (76.9)
Part Time	4 (26.7)	Part Time	0 (0.0)
Homemaker	0 (0.0)	Homemaker	3 (23.1)
**Estimated household income**		**Estimated household income**	
$29,999 and under	0 (0.0)	$29,999 and under	0 (0.0)
$59,999–$30,000	0 (0.0)	$59,999–$30,000	0 (0.0)
$79,999–$60,000	3 (20)	$79,999–$60,000	2 (15.0)
$80,000–$124,999	5 (33)	$80,000–$124,999	4 (31.0)
$125,000 and over	7 (47)	$125,000 and over	7 (54.0)
**Type of community of residence**		**Type of community of residence**	
City	10 (67.0)	City	9 (70.0)
Town	4 (27.0)	Town	2 (15.0)
Rural	1 (6.0)	Rural	2 (15.0)
**Highest level of education**		**Highest level of education**	
Community college/high school	2 (13.3)	Community college/high school	2 (15.4)
Bachelor's degree/undergraduate	8 (53.3)	Bachelor's degree/undergraduate	5 (38.5)
Master's degree	2 (13.3)	Master's degree	5 (38.5)
Degree in medicine	3 (20.0)	Degree in medicine	1 (7.6)
**Child demographics (Tx group;** ***N** =* **15)**		**Child demographics (Ctrl group;** ***N** =* **13)**	
**Child sex**		**Child sex**	
Male	7 (47.0)	Male	4 (30.0)
Female	8 (53.0)	Female	9 (70.0)
Child Mean Age in Years (*SD*)	8.47 (1.46)	Child Mean Age in Years (*SD*)	8.23 (1.54)
**Child's ethnic or cultural heritage**		**Child's ethnic or cultural heritage**	
White	11 (73.3)	White	9 (69.2)
Indigenous	1 (6.7)	Indigenous	2 (15.4)
Other	3 (20.0)	Other	2 (15.4)

Tx, Treatment; Ctrl, Control.

### Estimations for full-scale RCT

Enrollment for the current pilot RCT was 202 participants eligible to participate. Projected participant retention rate was 70% and the dropout rate was 30%. Our a priori sample size calculation for the future full-scale trial was made with the following parameters in G^*^Power: α error probability = 0.05, power = 0.80, effect size *f* = 0.3145 (i.e., $\eta_{p}^{2}$ = 0.09, medium). These parameters estimated that 82 participants would be required to observe a medium effect size per group for our main outcome measures (i.e., sleep habits; CSHQ and BRQ). Based on the 30% dropout rate, the overall final sample size that would be required for the future full-scale RCT to retain 41 participants per group after the intervention is approximately 118 participants enrolled for the pre-intervention period. As such, the final sample size to detect a medium effect of the intervention would be 82 participants overall.

### Preliminary effectiveness

***Subjective measures of sleep and behavioral functioning***. The data were screened before the ANCOVAs were conducted. The only assumption violated was that of equal variances as Levene's test was significant for the CSHQ and PISI variables. As such, robust ANCOVAs were conducted to determine whether results were maintained. The robust ANCOVAs produced the same null hypothesis test results as the original ANCOVAs (described below) and robust ANCOVA data are reported in the notes section of Table 3 for the CSHQ and PISI. As such, the original ANCOVA results are described and maintained in the body of Table 3.

**Table 3:** Analysis of covariance (ANCOVA) results for subjective data.

**Variable**		**Mean difference between baseline and follow-up**	**d*f***	***F***	***P***	**ω^2^**	**95% CI**	
							**Lower**	**Upper**
CSHQ^*^	Group assignment	−5.557	1	11.5	0.002	0.150	−8.936	−2.177
	Baseline		1	32.4	< 0.001	0.449		
	Residuals		25					
BRQ WD	Group assignment	2.443	1	10.1	0.004	0.178	0.863	4.023
	Baseline		1	15.3	< 0.001	0.277		
	Residuals		25					
BRQ WE	Group assignment	1.457	1	2.93	0.099	0.036	−0.296	3.209
	Baseline		1	24.14	< 0.001	0.436		
	Residuals		25					
PISI	Group assignment	−0.363	1	6.89	0.015	0.127	−6.002	−0.724
	Baseline		1	13.62	0.001	0.271		
	Residuals		25					
SDQ	Group assignment	−3.123	1	15.1	< 0.001	0.138	−4.779	−1.467
	Baseline		1	61.2	< 0.001	0.588		
	Residuals		25					

df = degrees of freedom; PISI, Pediatric Insomnia Severity Index; CSHQ, Children's Sleep Habits Questionnaire; BRQ WD, Bedtime Routines Questionnaire Weekday; BRQ WE, Bedtime Routines Questionnaire Weekend; SDQ, Strengths and Difficulties Questionnaire; **ω**^2^ were calculated to observe conservative effect sizes given the small sample size.

^*^Indicates that Levene's test was violated for CSHQ and PISI. Robustness checks were completed and results for CSHQ were as follows: mean difference = −5.56, CI 95%= −8.09 to −0.80, *p* = 0.019. Results for PISI were as follows: mean difference = −3.36, CI 95%= −7.25 to −0.49, *p* = 0.027. Both results indicate that results continue to be statistically significant when controlling for the violation of equality of variances for the CSHQ and PISI.

The ANCOVA conducted on the CSHQ total score outcome demonstrated significant changes in post-intervention sleep habits when controlling for pre-intervention scores and group assignment (i.e., waitlist control vs. treatment group), such that the CSHQ total score was lower before the intervention for those in the treatment group relative to the waitlist control group, with 15% of the variance in the CSHQ explained by group membership (*f* = 0.150). The same was true of the BRQ weekday post-intervention changes, such that the BRQ showed increased scores of consistency for those in the treatment group. Approximately 17% of the variance in the BRQ weekday post-intervention changes were explained by group membership (*f* = 0.178). Significant differences were not found for the BRQ weekend scores as weekend consistency did not improve in the treatment group relative to the waitlist control group. In terms of insomnia severity scores measured by the PISI, treatment group post-intervention scores were significantly lower after the intervention when controlling for pre-intervention scores with a small effect. Finally, behavioral functioning, as measured by SDQ scores, was significantly lower after the intervention when controlling for pre-intervention scores (i.e., daytime functioning improved) in the treatment group with a small effect.

Two of the subjective measures examined have established clinical cut-off scores, specifically, the CSHQ and the SDQ. The CSHQ has a clinical cut-off score of 41, with scores higher than 41 indicating significant sleep disturbance (Owens et al., 2000a,b). For the SDQ, the clinical cut-off score is 17, with scores higher than 17 indicating clinically elevated behavioral concerns (Bryant et al., 2020). Our CSHQ results demonstrated that before the intervention, our treatment group had significant sleep disturbance (*M* = 49.0, SD = 4.38) that was at the cut-off score after the intervention (*M* = 41.7, SD = 3.75). The same was not true of our control group, who had significant sleep disturbance before the intervention (*M* = 51.8, SD = 8.31) and after the intervention (*M* = 49.2, SD = 7.84). Before the intervention, 100% of participant scores for the CSHQ were above the clinical cut-off of 41, and after the intervention 54% were above the cut-off for the treatment group. For the SDQ, our results followed a pattern similar to that of the CSHQ results such that our treatment group had clinically elevated behavioral symptoms before the intervention (*M* = 18.8, SD = 3.59) that was at the cut-off score after the intervention (*M* = 17.3, SD = 3.82). Again, as with the CSHQ results, our control group did not have the same pattern as the treatment group, as their pre-intervention scores (*M* = 18.5, SD = 5.46) and post-intervention scores (*M* = 20.3, SD = 3.90) were both clinically elevated. Before the intervention, 77% of participant scores for the SDQ were above the clinical cut-off of 17, and after the intervention, 54% were above the cut-off for the treatment group. All other subjective sleep outcome means and standard deviations from before the intervention to after the intervention are presented in Table 4.

**Table 4:** Baseline to follow-up descriptives for subjective and objective sleep variables.

**Subjective**	**Baseline**				**Follow-up**			
	**Treatment**		**Waitlist control**		**Treatment**		**Waitlist control**	
**Variable**	***M***	***SD***	***M***	***SD***	***M***	***SD***	***M***	***SD***
CSHQ	49.0	4.3	51.8	8.3	41.7	3.7	49.2	7.8
BRQ WD	21.2	2.2	21.0	2.9	22.8	1.6	20.3	3.0
BRQ WE	20.4	3.1	18.9	4.2	21.8	1.7	19.5	3.7
PISI	15.4	3.9	16.0	4.7	11.2	4.9	14.9	3.2
SDQ	18.8	3.5	18.5	5.4	17.3	3.8	20.3	3.9
**Objective**								
SEF	84.9	3.5	75.8	17.1	85.4	1.6	83.5	5.9
SOL	34.2	53.1	17.3	17.0	6.89	6.2	6.67	8.5

CSHQ, Children's Sleep Habits Questionnaire; BRQ WD, Bedtime Routines Questionnaire Weekday; BRQ WE, Bedtime Routines Questionnaire Weekend; SDQ, Strengths and Difficulties Questionnaire; SEF, sleep efficiency; SOL, sleep onset latency.

***Objective sleep measure (actigraphy)***. A detailed breakdown of the results for each variable of the objective sleep measure (actigraphy) is provided in Tables 5 (ANCOVA results) and 3.4 (*M* and *SD* from before the intervention to after the intervention) and briefly described in this section. Before conducting the ANCOVAs for SOL and SEF it was determined that both violated the assumption of normality. Given this, robust ANCOVAs were conducted with no changes in the results of the ANCOVA, and as such the results of the ANCOVAs are reported. No effect of the intervention was observed for either SOL or SE from before the intervention to post-intervention for either group.

**Table 5:** Analysis of covariance (ANCOVA) for objective sleep variables.

**Variable**		**Mean difference**	**d*f***	***F***	***p***	**ω^2^**	**95% CI**	
							**Lower**	**Upper**
SEF	Group assignment	2.5414 (percentage)	1	1.003	0.336	0.000	−2.988	8.071
	Baseline		1	0.437	0.521	−0.039		
	Residuals		12					
SOL	Group assignment	0.3241 (min)	1	0.00647	0.937	−0.074	−8.456	9.105
	Baseline		1	0.38516	0.546	−0.046		
	Residuals		12					

df, degrees of freedom; SEF, sleep efficiency; SOL, sleep onset latency; CI, confidence interval; **ω**^2^ were calculated to observe conservative effect sizes given the small sample size. Note that ω^2^ statistics can sometimes be negative, unlike $\eta_{\text{p}}^{2}$. These should be interpreted as an effect size of zero (i.e., no variance explained), though statistical recommendations suggest reporting the negative values for use in meta-analyses (Okada et al., 2017).

## Discussion

The goal of this pilot RCT study was to estimate parameters for a full-scale RCT and to investigate the preliminary effectiveness of the recently revised ABCs of SLEEPING intervention by evaluating changes in school-age children's sleep and daytime functioning. Sleep was measured both subjectively (parent-completed questionnaire) and objectively (actigraphy). Overall, our parent sample mostly consisted of highly educated White mothers, and their children were mostly White females and had high parent-reported pre-intervention insomnia symptom severity. As for preliminary effectiveness, we noted improvements in sleep habits as measured by the CSHQ and with bedtime routines (BRQ) for weekdays (not weekends), insomnia symptom severity (PISI), and in daytime functioning (SDQ). While clinically elevated before the intervention, after the intervention period our treatment group's sleep disturbance score (CSHQ) and its behavioral functioning (SDQ) were at the cut-off determined to be clinically elevated while the control group remained clinically elevated. No significant effects of the intervention were found for objective sleep data (actigraphy).

### Estimations for a future full-scale RCT

Important feasibility data (i.e., recruitment potential, retention, dropout rate, sample size estimation) was collected in this pilot RCT to identify the parameters needed for a future full-scale RCT, which is the next step for future research evaluating the ABCs of SLEEPING intervention. This addressed our first research question focused on making estimates for a future full-scale RCT. Note that estimates do not adjust for demographic covariates like age and sex. Analysis of our data revealed 552 participants clicked into REDCap prior to completing the EQ. Enrollment in the current pilot RCT consisted of 202 participants. This study's participant retention rate was 70%, and the dropout rate was 30%. A priori sample size calculations revealed 82 participants (41 per group) would be required to detect a medium effect size for our sleep habit measures (CSHQ and BRQ). Based on the participant dropout rate, the estimated sample size for the future-scale trial is approximately 118 participants needed before the intervention to retain 41 participants per group by the post-intervention period.

### Subjective measures of sleep

Our second research question focused on understanding changes in parent-reported sleep habits, insomnia severity, and behavioral functioning of their children in response to the use of the ABCs of SLEEPING intervention. Given that the intervention aims to provide healthy sleep practice information so parents can adjust or continue their child's sleep habits, our primary outcome variables were the CSHQ total sleep score and BRQ weekday and weekend consistency of sleep habits. Our results revealed gains in the treatment group, relative to the waitlist group, for the CSHQ and for the BRQ weekday consistency with small effect sizes. No statistically significant improvements were noted for BRQ weekend consistency. This demonstrates that the intervention may have success in adjusting school-age children's sleep habits, specifically during weekdays. Although clinical significance cannot be asserted as a result of our analyses, it was promising to see a reduction in the treatment group approaching the nonclinical level on the measures of sleep habits, insomnia symptoms, and daytime behavioral functioning.

There were no statistically significant changes in weekend sleep habits. Weekday to weekend sleep discrepancies have been well documented in the literature (Sun et al., 2019; Merdad et al., 2014; Becker et al., 2017; Chien et al., 2019). Lack of change during weekends may be explained by nonroutine events typically occurring during this period of time (e.g., family/sporting events, holiday) and more routine events occurring during weekdays (e.g., school attendance). Improved behavioral functioning for the treatment group is unsurprising since the impact of insomnia on behavioral functioning is well documented in the literature (Calhoun et al., 2017; Armstrong et al., 2014; de Zambotti et al., 2018; Haynes et al., 2011). Additionally, our behavioral functioning results are consistent with that of previous research evaluating insomnia treatments via a RCT that also found improved behavioral functioning (Hiscock et al., 2007). Although our data cannot confirm this, it may be possible that the ABCs of SLEEPING indirectly improves behavioral functioning by directly improving sleep habits such as bedtime routines.

### Objective measure of sleep

For our third research question, no effect of the intervention was observed on the objective sleep measure (actigraphy, SE, and SOL), despite subjective reports of improvements in sleep habits and insomnia severity. This is unsurprising since subjective (e.g., self-reported) and objective measures (e.g., actigraphy) have historically shown mixed results in the literature in both adult and child populations, with some research noting only moderate associations between subjective measures and objective measures of sleep duration (Lauderdale et al., 2008; Arora et al., 2013; Girschik et al., 2012; Silva et al., 2007), while others have noted weaker associations (Alfano et al., 2007; Silva et al., 2007; Benz et al., 2023). This is a common finding across RCTs examining sleep interventions broadly. A recent review conducted by Lah and Cao (2024), which included nine RCTs with older school-age children and adolescents, also found differences in subjective measures but not objective measures of sleep (e.g., De Bruin et al., 2015a,b; Corkum et al., 2016, as outlined in Table 4 of their manuscript). It has been proposed that discrepancies between actigraphy and sleep questionnaire data may be influenced by factors such as memory, experiences and psychosocial pressures, recall and/or sleep-related bias, and vague or inaccurate reporting (Akram et al., 2023; Iwasaki et al., 2010; Werner et al., 2008; Salbach-Andrae et al., 2009).

## Limitations and strengths

This study had a number of strengths. Specifically, the RCT methodology used allowed for increased control when measuring sleep outcomes (e.g., randomization, controlling for sleep medication). Additionally, both subjective and objective measures were used to examine sleep. Lastly, pilot studies themselves provide helpful insights, including the opportunity to refine and develop new methodological practices when planning for a future full-scale RCT (Leon et al., 2011). For example, we will be better able to understand and plan for the screening and recruitment process (e.g., total number of participants to be recruited and participant retention) and may incorporate new recruitment strategies to increase diversity.

This study was not without limitations. Importantly, the current pilot RCT study examined effectiveness in a limited way (i.e., preliminary effectiveness). Specifically, our sample size was limited, especially for our objective measurement of sleep. Given this, the data should be interpreted with caution. Also, our sample consisted of mostly White and highly educated biological mothers and as such cannot be generalized to other populations. Moreover, we do not have information on why some parents chose not to participate, so there may be some selection bias. Additionally, children with existing mental health diagnoses (including neurodevelopmental disorders) were excluded, which limits generalizability to these populations. Also, we did not examine the fidelity of the intervention, so it is not clear to what extent families used the intervention. Due to a technical software error, user statistics were not saved, and as such we cannot examine these to determine the use of the app (e.g., number of logons, time spent on the app). Additionally, instructions regarding intervention use were not formalized, and no reminders were sent out about the intended use of the intervention. We also did not evaluate whether the effects of the intervention were maintained after a longer follow-up period. As such, it is not clear whether changes in our subjective sleep measure variables were short term or would be maintained long term. Also, in this study, inclusion criteria were very broad (e.g., no requirement to meet diagnostic criteria of insomnia but just to display symptoms of insomnia). Last, the current study examined only two actigraphy sleep variables (SOL and SEF), but others could have been explored (e.g., total sleep time, wake after sleep onset). While we did not identify changes in SOL and SEF, it is possible that a treatment effect may have been found on other actigraphy variables not analyzed in this study. The previously noted limitations should be addressed before future full-scale testing for the ABCs of SLEEPING intervention. In particular, the next study should ensure that a more diverse sample is recruited and should incorporate methodological changes to ensure increased confidence when drawing conclusions from the data (e.g., fidelity checks, collection of user statistics, follow-up period examining for maintained effects, increased sample size).

## Future research

The next step for future research examining the ABCs of SLEEPING intervention is to conduct a full-scale RCT that incorporates knowledge acquired through this research (e.g., more representative sample, refined methodological practices). Additionally, further investigation could help to clarify the specific components, or “active ingredients,” that drive improvements in sleep outcomes, whether these relate to changes in parental engagement, bedtime routines, and/or other behavioral mechanisms. Moreover, it would be important to collect information about previous sleep interventions the parents had implemented to better understand the context in which the current intervention was successful. Further, developing and evaluating a version of this intervention for parents of children with neurodevelopmental disorders would be important given the high prevalence and significant impact of sleep problems in this population. In future research, it would be helpful to recruit a more diverse sample, including equity-deserving families, to test whether the intervention is helpful to these families. Lastly, testing the intervention with parents of children who meet criteria for insomnia would be important to confirm that these children benefit from this app.

## Conclusion

In conclusion, parents using the ABCs of SLEEPING intervention reported improvements in their child's post-intervention sleep habits, weekday bedtime routines, insomnia severity, and behavioral functioning, relative to parents of children who did not have access to the ABCs of SLEEPING app. No changes were found in weekend bedtime routines or on actigraphy-measured SE or SOL. If future large-scale testing confirms the effectiveness of the intervention, the ABCs of SLEEPING intervention has the potential to address some of the key accessibility barriers experienced by families through the provision of an accessible, scalable, and evidence-based sleep intervention app to promote sleep health in children.

## Data Availability

The original contributions presented in the study are included in the article/supplementary material, further inquiries can be directed to the corresponding author.
